# Infections and obsessive-compulsive disorder - results from a systematic review and meta-analysis

**DOI:** 10.1192/j.eurpsy.2023.529

**Published:** 2023-07-19

**Authors:** N. Descalço, R. D. Gomes, A. Maia, B. Barahona-Corrêa, A. Oliveira-Maia, J. Oliveira

**Affiliations:** ^1^Neuropsychiatry Unit, Champalimaud Research and Clinical Centre; ^2^NOVA Medical School, NOVA University of Lisbon, Lisbon; ^3^Psychiatry and Mental Health Department, Hospital Garcia de Orta, Almada; ^4^Department of Psychiatry and Mental Health, Centro Hospitalar Lisboa Ocidental, Lisbon, Portugal

## Abstract

**Introduction:**

Obsessive-compulsive disorder (OCD) is a psychiatric disorder affecting 1.3% of the population worldwide where both genetic and environmental factors, such as perinatal events and neuroinflammation, are thought to contribute to the etiology of the disorder. In the past, the description of clinical entities such as Pediatric Autoimmune Neuropsychiatric Disorder Associated with Streptococcal Infections (PANDAS), in which an acute neuropsychiatric syndrome with prominent obsessive-compulsive features emerges in children infected with *group-A beta-hemolytic streptococcus* (GABHS), sparked the hypothesis that infections may be a risk-modifying factor for the development of OCD. Along with streptococcal infections, other pathogens such as *Toxoplasma gondii* have been implicated in the pathophysiological models of the disorder, although causal associations have not been established for any of beforementioned pathogens.

**Objectives:**

To perform a systematic review and meta-analysis about the presence of biological evidence of infection in patients diagnosed with OCD.

**Methods:**

We conducted a systematic review and a meta-analysis (PROSPERO registration CRD42021223415) by performing a standardized electronic database search in MEDLINE/PubMed, Web of Science, Embase and Scopus. Search was conducted on 17/10/2022. Eligible papers included case-control and cohort studies using a comparator group, that tested for specific biomarkers providing evidence of infection in patients diagnosed with OCD; exclusion criteria included studies without quantitative or qualitative measures of infection, case reports, systematic or scope reviews, and animal studies. Selection process was conducted according to PRISMA 2020 statement guidelines. Study quality was assessed through Newcastle-Ottawa Quality Assessment Scale.

**Results:**

We identified 8911 records through the search after duplicate removal. A total of 22 studies met inclusion criteria after selection process, and 15 were eligible for meta-analysis. Most evidence concerned *Toxoplasma gondii* (10 studies), and patients with OCD appear to have higher odds of being infected compared to controls, with a meta-analytic odds ratio of 2.39 (95% IC 1.60-3.58), when comparing 467 patients with 5411 controls. However, most studies were methodologically heterogeneous, which compromises the interpretation of meta-analytic results. Information regarding other agents, including GABHS, *Borna disease virus* and *Toxocara canis* was gathered but due to an insufficient number of papers it was not possible to perform a meta-analysis for each of them.

**Conclusions:**

Our work suggests that albeit exhaustively reported in the literature, there is no strong evidence of the over-representation of biomarkers of infection in patients with OCD compared to control volunteers. Methodologically robust studies are needed to further test this hypothesis.

**Disclosure of Interest:**

None Declared

